# ASSOCIATIONS OF PREVENTION FOCUS TRAITS WITH SEDENTARY BEHAVIOR IN COMMUNITY-DWELLING OLDER ADULTS

**DOI:** 10.1093/geroni/igae098.3545

**Published:** 2024-12-31

**Authors:** Jethro Raphael Suarez, Amber Blount, Joon-Hyuk Park, Ladda Thiamwong

**Affiliations:** University of Central Florida; University of Central Florida, Orlando, Florida, United States; University of Central Florida, Orlando, Florida, United States; University of Central Florida, Orlando, Florida, United States

## Abstract

Sedentary behavior is prevalent among older adults and can increase the risk of developing health conditions, such as muscle atrophy and heart disease. Beyond physical factors, it is important to determine mental factors that could be associated with excessive sedentary behavior. The Regulatory Focus Questionnaire (RFQ) is an 11-item instrument that allows an individual to self-report the frequency of occurrences of specific events in their life. The RFQ yields 2 separate scores: a promotion focus score and prevention focus score. A higher promotion focus score describes an individual who is goal-motivated, open to change, and willing to take risks. A higher prevention focus score describes an individual who is motivated by avoiding losses, more cautious, prefers constant routine, detail-oriented, and avoids risks. A cross-sectional study was conducted with 94 community-dwelling older adults aged 61 to 89 years (84 women, 10 men; mean age = 74.87 ± 6.76 years) to determine the relationship between total measured sedentary behavior and promotion and prevention focus, respectively. Sedentary behavior was objectively measured for each participant using the ActiGraph GT9X Link wireless activity monitor over 7 consecutive days. Kendall’s Tau test revealed that sedentary behavior had a significant positive correlation with prevention focus scores (p = 0.00819, τ = 0.194), but not with the promotion focus scores (p = 0.647, τ = -0.0335). These findings suggest that certain personality traits, such as being loss-avoidant, detail-oriented and less accepting of change, can be associated with older adults that engage in increased sedentary behavior.

